# Stearoyl-CoA desaturase-1: a potential therapeutic target for neurological disorders

**DOI:** 10.1186/s13024-024-00778-w

**Published:** 2024-11-19

**Authors:** Melanie Loix, Sam Vanherle, Marta Turri, Stephan Kemp, Karl J. L. Fernandes, Jerome J. A. Hendriks, Jeroen F. J. Bogie

**Affiliations:** 1 Department of Immunology and Infection, Biomedical Research Institute, Hasselt University, Diepenbeek, Belgium; 2 University MS Center Hasselt, Pelt, Belgium; 3 Research Center on Aging, CIUSSS de l’Estrie-CHUS, Sherbrooke, Canada; 4 Laboratory Genetic Metabolic Diseases, Department of Laboratory Medicine, Amsterdam Neuroscience, Amsterdam UMC Location University of Amsterdam, Amsterdam Gastroenterology Endocrinology Metabolism, Amsterdam, NH Netherlands; 5 Department of Medicine, Faculty of Medicine and Health Sciences, Université de Sherbrooke, Sherbrooke, Canada

**Keywords:** Neurodegenerative disorders, Fatty acid metabolism, Stearoyl-CoA desaturases, Cellular and molecular dysfunction

## Abstract

Disturbances in the fatty acid lipidome are increasingly recognized as key drivers in the progression of various brain disorders. In this review article, we delve into the impact of Δ9 fatty acid desaturases, with a particular focus on stearoyl-CoA desaturase-1 (SCD1), within the setting of neuroinflammation, neurodegeneration, and brain repair. Over the past years, it was established that inhibition or deficiency of SCD1 not only suppresses neuroinflammation but also protects against neurodegeneration in conditions such as multiple sclerosis, Alzheimer’s disease, and Parkinson’s disease. This protective effect is achieved through different mechanisms including enhanced remyelination, reversal of synaptic and cognitive impairments, and mitigation of α-synuclein toxicity. Intriguingly, metabolic rerouting of fatty acids via SCD1 improves the pathology associated with X-linked adrenoleukodystrophy, suggesting context-dependent benign and harmful effects of SCD1 inhibition in the brain. Here, we summarize and discuss the cellular and molecular mechanisms underlying both the beneficial and detrimental effects of SCD1 in these neurological disorders. We explore commonalities and distinctions, shedding light on potential therapeutic challenges. Additionally, we touch upon future research directions that promise to deepen our understanding of SCD1 biology in brain disorders and potentially enhance the clinical utility of SCD1 inhibitors.

## Introduction

Fatty acids (FAs) play a crucial role in supporting various cellular and tissular functions, encompassing the synthesis of membranes, signal transduction, energy storage, and protein modifications. These multifaceted processes necessitate the presence of a diverse array of lipids, for which an intricate network of metabolic pathways has evolved to facilitate their formation. The two predominant types of FAs found in mammalian organisms are saturated FAs (SFAs) and monounsaturated FAs (MUFAs). Among these, MUFAs are particularly favored as substrates for the synthesis of complex lipids species such as phospholipids, triglycerides (TGs), and cholesteryl esters (CEs), which represent major building blocks of biological membranes, as well as a cellular energy source and signaling molecules. Maintaining a balanced ratio of SFAs and MUFAs is crucial for proper cell physiology, as disturbances in this balance can significantly impact cellular function in health and disease, in particular in the lipid-rich central nervous system (CNS) [1].

## Function and expression of Δ9 fatty acid desaturases

Intracellular levels of SFAs and MUFAs are controlled by a family of Δ9 FA desaturases, so called stearoyl-CoA desaturases (SCDs). SCDs are four-span transmembrane proteins that are localized in the endoplasmic reticulum (ER) and function to introduce a cis-double bond at the Δ9 position of FAs. Mechanistically, the double bond formation is catalyzed by a diiron center in SCDs, where Fe^2+^ interacts with molecular oxygen to extract hydrogen atoms from FAs. The recovery of the diiron center is facilitated by an electron transfer chain comprising NADH, cytochrome b5 reductase, and cytochrome b5 [2]. According to the prevailing hypothesis, the desaturation reaction catalyzed by SCDs follows a specific pattern, involving the sequential removal of hydrogen atoms from the C-9 and C-10 position of palmitic acid (C16:0) and stearic acid (C18:0) [3, 4]. This reaction leads to the formation of palmitoleic acid (C16:1) and oleic acid (OA, C18:1).

SCDs are widely distributed in all organisms, spanning from unicellular organisms to vertebrates. In *Saccharomyces cerevisiae*, there is only one SCD orthologue, known as Ole1, which is localized to the ER [5]. In contrast, *C. elegans* and *D. melanogaster* have a greater number of SCD orthologues: three in *C. elegans* (Fat-5, Fat-6, and Fat-7) and two in *D. melanogaster* (Desat1 and Desat2) [6, 7]. Similarly, vertebrates, including mice and humans, express multiple isoforms that share remarkable similarities in their biochemical activity and sequence [8–12]. Mice, for example, contain four SCD isoforms (SCD1–4) that are expressed in a tissue-specific manner, with SCD1 being present in all tissues, in particular in lipogenic tissues, and SCD2, SCD3 and SCD4 being highly abundant in brain, skin, and heart, respectively. Humans, on the other hand, have two SCD isoforms, SCD1 and SCD5. Similar to mice, human SCD1 is ubiquitously expressed, with particularly high levels in lipogenic tissues, while human SCD5 is predominantly expressed in the brain and pancreas. Among these isoforms, SCD1 has been most extensively studied and implicated in a wide range of inflammatory and metabolic diseases, including cancer, obesity, cardiac pathologies, atherosclerosis, and autoimmune disorders (reviewed in [13–15]). In this review, we aim to delve into the role of SCD1 in neurodegenerative and neuroinflammatory disorders, explore its therapeutic potential in these pathological conditions, and identify potential challenges for its use as a drug target.

## SCD1 in neurological disorders

### X-linked adrenoleukodystrophy

X-linked adrenoleukodystrophy (X-ALD) is a neurometabolic disease caused by pathogenic variants in the ATP binding cassette (ABC) subfamily D member 1 (*ABCD1*) gene, which is located on the X chromosome (Xq28) and encodes the peroxisomal transporter of CoA-activated saturated very long–chain FAs (VLCFAs; ≥22 C atoms). Pathogenic variants in *ABCD1* lead to impaired import of VLCFAs into peroxisomes, resulting in reduced peroxisomal VLCFA β-oxidation and their accumulation in plasma and tissue, including adrenal glands, spinal cord, and brain (reviewed in [16]). In childhood, males with X-ALD often develop progressive damage of the adrenal cortex and wide-spread detoriation of the myelin sheath surrounding the axons in cerebral white matter, so called demyelination. In adulthood, both males and females develop a slowly progressive myeloneuropathy [17]. There are several theories on how the accumulation of VLCFAs leads to cellular and tissular damage. These mechanisms range from VLCFAs disrupting membrane phospholipid function and stability, to enhancing the production of reactive oxygen species and exerting direct cytotoxic effects (reviewed in [18]).

**Fig. 1:**
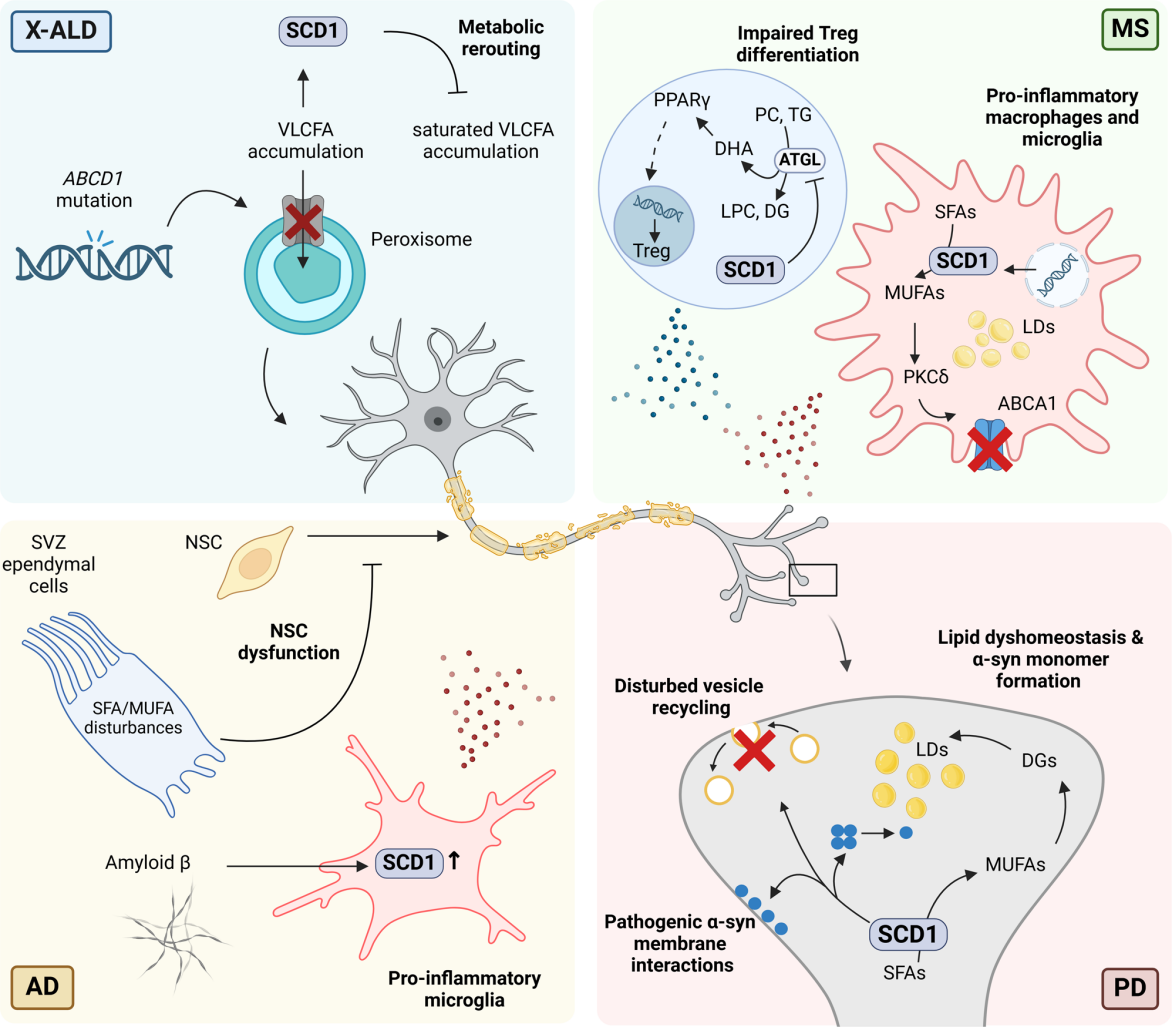
SCD1 is a driver of immune, glia, and neuronal physiology in neurodegenerative disorders such as X-ALD, MS, AD, and PD. In X-linked adrenoleukodystrophy (X-ALD, top left), SCD1 plays a crucial protective role by redirecting saturated to monounsaturated very long–chain fatty acids (VLCFAs), thereby mitigating VLCFA-induced lipotoxicity. However, contrary to X-ALD, heightened activity and abundance of SCD1 drive disease progression in multiple sclerosis (MS), Alzheimer’s disease (AD), and Parkinson’s disease (PD). In MS (top right), SCD1-produced monounsaturated FAs (MUFA) destabilize plasma membrane ATP-binding cassette transporter ABCA1 in a PKCδ-dependent manner, leading to intracellular accumulation of highly inflammatory free cholesterol in foamy myelin-containing macrophages and microglia. Moreover, SCD1 acts as a brake on Treg differentiation, exacerbating neuroinflammation by suppressing adipose triglyceride lipase (ATGL)-dependent release of docosahexaenoic acid (DHA) and subsequent peroxisome proliferator-activated receptor γ (PPARγ) activation. In AD (bottom left), disrupted saturated FAs (SFA)/MUFA ratios disrupt ependymal cell function in the subventricular zone (SVZ), impairing neural stem cell (NSCs) physiology (e.g. proliferative deficits), a hallmark of AD. Additionally, SCD1 exacerbates the formation of a disease-promoting microglial phenotype upon amyloid β engulfment. In PD (bottom right), SCD1 is implicated in α-synuclein (α-syn)-induced vesicle trafficking deficits and pathogenic interactions with membranes. Increased SCD1-generated oleic acid (OA) enhances ER-mediated α-syn toxicity, potentially through incorporation into diglycerides (DGs) and lipid droplets (LDs), while also promoting the formation of aggregation-prone α-syn monomers, thus heightening α-syn membrane association and neurotoxicity

Given that cellular accumulation of VLCFAs is the primary pathological hallmark of X-ALD, therapeutic strategies focused on normalizing VLCFA levels have been regarded as central to treatment approaches. However, emerging evidence indicates that VLCFA quality, rather than or in addition to quantity, underpins their harmful impact in X-ALD. By using human X-ALD fibroblasts and a zebrafish *Abcd1* mutant model, metabolic rerouting of saturated to monounsaturated VLCFAs by SCD1 was found to attenuate lipid toxicity (Fig. 1) [19]. Specifically, the authors demonstrated that enhancing SCD1 expression and activity using an agonist for liver X receptor (LXR), a nuclear receptor which senses cholesterol and regulates the expression of lipid metabolism genes including *Scd1* [20–23], partially normalized the accumulation of toxic saturated VLCFA levels in X-ALD fibroblasts and male *Abcd1-deficient (Abcd1*^*–/y*^*) mice* [19]. Vice versa, CRISPR knockout of *Scd1* mimicked the motor phenotype observed in *Abcd1* zebrafish mutants. These findings are consistent with exposure to saturated, but not monounsaturated, VLCFAs inducing ER stress and perturbing oxidative stress homeostasis and mitochondrial function in human X-ALD fibroblasts [24–27]. Notably, analysis of liver histology of wild-types and *Abcd1*^*–/y*^ mice treated with the LXR agonist T0901317 also showed adverse effects, including liver steatosis, cell ballooning, and active inflammation [28]. While this study identified the importance of SCD1 in preventing the toxic accumulation of saturated VLCFAs, more research is warranted to confirm the therapeutic potential of increasing SCD1 expression in X-ALD and to assess the suitability of LXR agonists to do so. For example, it would be valuable to assess whether treatment with tissue- or isoform-specific LXR agonists [28], or other SCD1 modulators with reduced toxicity, can impact axonal degeneration in X-ALD without causing adverse side effects in the liver. More research is also needed to elucidate the molecular mechanisms underpinning the impact of LXR modulators on VLCFA quality. While LXR activation is indeed a driver of SCD1 expression, the endogenous LXR agonist 25-hydroxcholesterol is also reported to reduce saturated VLCFAs in X-ALD fibroblasts and oligodendrocytes by decreasing the expression of VLCFA elongase 1 (ELOVL1) [29]. Further, LXR activation can reduce ABCD2 and ABCD3 abundance, which represent essential compensatory enzymes for lack of ABCD1 [30]. Hence, the protective impact of LXRs on SCD1 abundance may be nullified by a simultaneous decrease in ABCD2 and ABCD3 activity. Notably, alongside increasing ABCD2 and ABCD3 [31], fibrates increase SCD1 expression through activation of peroxisome proliferator-activated receptor (PPAR) α and sterol regulatory element-binding protein (SREBP)1c [32], and therefore could represent an alternative therapeutic approach to simultaneously promote peroxisomal β-oxidation of VCLFAs and the metabolic rerouting of saturated to monounsaturated VLCFAs in X-ALD [33]. Finally, cell type-dependent and conditional differences in SCD1 expression and activity, as well as the SFA/MUFA balance, might well impact the functional properties of fibroblasts, neurons, and glial cells in X-ALD differently. In support of this notion, while being protective in X-ALD fibroblasts, increased SCD1 activity in multiple sclerosis (MS), Alzheimer’s disease (AD), and Parkinson’s disease (PD) is associated with the formation of disease-promoting microglia and neuronal dysfunction (see Sect. 3.2–3.4). Hence, in-depth profiling of cellular differences in response to changes in the abundance of SFA, MUFA, and VLCFAs, as well as the activity of ABCD1 and SCD1, is likely to provide increased insight into X-ALD disease pathology.

### Multiple sclerosis

MS is a neurodegenerative disease of the CNS that is fueled by an unequivocal autoimmune response directed against CNS-derived antigens. While genetic predisposition accounts for part of the disease risk [34], a range of lifestyle and environmental factors also play a critical role in disease progression (reviewed in [35]). Key players in the immune response in MS include autoreactive T cells which cross the blood-brain barrier (BBB) and trigger inflammation. This leads to the CNS infiltration and activation of additional immune cells, including macrophages and microglia, resulting in demyelination and axonal loss [36]. While endogenous CNS repair processes such as remyelination are apparent in early MS lesions, they frequently fail in progressive forms of MS. This has profound pathophysiological consequences as sustained loss of myelin disrupts axonal integrity and increases the susceptibility of axons to inflammatory mediators [37]. With neurodegeneration standing as the primary catalyst for MS disease symptoms, these studies accentuate the significance of unraveling the cellular and molecular drivers of failure of CNS repair in MS.

Growing evidence from preclinical models that mimic autoimmunity-mediated demyelination and remyelination highlights that imbalances in FA metabolism and levels drive MS disease pathology by regulating the inflammatory and regenerative properties of immune cells (reviewed in [1]). Of particular interest, SCD1 abundance and activity are highly elevated in macrophages and microglia in active brain lesions of MS patients, and loss and inhibition of SCD1 improves brain repair in toxin-induced ex vivo and in vivo models for demyelination [38]. Mechanistically, SCD1-generated MUFAs were demonstrated to reduce the surface abundance of cholesterol efflux transporter ATP-binding cassette transporter A1 (ABCA1) in a protein kinase C (PKC) δ-dependent manner. Loss of ABCA1 enhanced the intracellular accumulation of highly inflammatory free cholesterol in foamy myelin-containing macrophages and microglia, thereby promoting the induction of an inflammatory, disease-promoting phagocyte phenotype (Fig. 1). These findings are consistent with previous studies showing that MUFAs can destabilize membrane ABCA1 and inhibit ABCA1-mediated cholesterol efflux [39–42]. The authors further showed that activation of LXRs by myelin-derived cholesterol, LXRβ in particular, increased SCD1 expression in macrophages and microglia after sustained intracellular accumulation [38, 43]. Collectively, these findings provide a molecular rationale for the progressive nature of demyelinating lesions in MS and identify SCD1 as a promising therapeutic target to promote remyelination. Nevertheless, in light of the deleterious consequences associated with heightened SCD1 activity in foam cells, further investigation is essential to elucidate the underlying molecular basis for the escalated SCD1 activity in these cells. In particular, the findings emphasize the crucial role of continuous endogenous LXRβ activation and subsequent SCD1 induction in driving the formation of inflammatory foam cells in MS, despite the prevailing assumption that LXR activation suppresses the formation of inflammatory macrophage and microglia subsets (reviewed in 44). While these findings could be contingent on specific cellular and environmental differences, they align with existing evidence that prolonged LXR activation, in contrast to short-term activation, can elicit inflammatory responses in human macrophages [43]. Furthermore, since the expression levels of *Scd1* transcripts are notably elevated in mature myelin-forming oligodendrocytes compared to their precursor cells [45], it is imperative to undertake a more in-depth investigation into the consequences of inhibiting SCD1 in this specific cell population as well.

Alongside suppressing remyelination by promoting the induction of an inflammatory phagocyte phenotype, more recent findings indicate an important role for SCD1 in autoimmune-mediated demyelination in MS as well, with patient-derived T cells displaying enhanced SCD1 activity [46]. Pharmacological inhibition and genetic deficiency of SCD1 was further found to reduce autoimmune-mediated demyelination in the experimental autoimmune encephalomyelitis (EAE) model, a well described animal model for MS that mimics autoimmune-mediated degenerative events in the CNS [47]. Mechanistically, loss and inhibition of SCD1 primed naïve CD4^+^ T cells to differentiate into immunosuppressive regulatory T cells (Tregs). Guided by RNA sequencing and lipidomics analysis, absence of SCD1 in T cells was found to enhance hydrolysis of TGs and phosphatidylcholine through adipose triglyceride lipase (ATGL). This process culminated in the release of docosahexaenoic acid (DHA), an omega-3 PUFA, which, in turn, augmented Treg differentiation and diminished CNS pathology in EAE mice by activating the nuclear receptor PPARγ (Fig. 1) [46]. Intriguingly, the latter finding suggest a reciprocal interaction between SCD1 and PPARγ, where *SCD1* is not merely a responsive gene of PPARγ but also contributes to the continuous activation of PPARγ [48]. All in all, this study provides evidence that SCD1 acts as an endogenous brake on the differentiation of Tregs, thereby likely contributing to autoimmune-mediated demyelination and neuroinflammation in the EAE model. Although these findings align with previous observations showing that (1) absence of SCD1 heightens splenic follicular Tregs after influenza immunization [49], (2) ATGL favors the hydrolysis of TGs and phosphatidylcholine rich in PUFAs [50], (3) DHA is a potent endogenous ligand for PPARγ [51], and (4) both DHA and PPARγ promote Treg differentiation and suppress neuroinflammation [51–58], several unanswered questions persist. For example, in contrast to promoting the differentiation of immunomodulatory Tregs in the EAE model, absence of SCD1 has also been reported to enhance the colitogenic potential of *Scd1*^−/−^ CD4^**+**^CD25^−^ T cells when transplanted into *Rag1*^−/−^ mice [59], an immunodeficient mouse model lacking mature T and B cells. Deficiency of *Scd1* led to elevated levels of SFAs, resulting in an enhanced secretory profile of effector T cells and, unexpectedly, an increased cellular membrane fluidity. While differences in the experimental animal models used may account for this discrepancy, it also raises the intriguing question of whether increased Treg differentiation serves as a protective response against the concurrent induction of the colitogenic potential in T cells deficient in *Scd1*. In addition, the precise molecular mechanisms driving increased ATGL-dependent release of DHA in T cells lacking SCD1 await further elucidation. While it could signify a compensatory mechanism aimed at maintaining sufficient cellular MUFAs, ATGL-mediated liberation of free DHA could also ensue to safeguard T cells against ER stress and cellular inflammation triggered by elevated SFA levels, potentially through the activation of PPARγ [60, 61]. Supporting this hypothesis, dietary supplementation with ω-3 PUFAs has been shown to mitigate the side-effects associated with SCD1 inhibition in *Ldlr*^*−/−*^*ApoB*^*100/100*^ mice, including the acceleration of atherosclerosis, lipoprotein abnormalities, and heightened toll-like receptor 4 (TLR4) sensitivity [62]. All in all, these intriguing discrepancies and molecular mechanisms associated with SCD1 deficiency merit further exploration.

### Alzheimer’s disease

AD is a neurodegenerative disorder characterized by a progressive decline in cognitive function, driven by complex pathological mechanisms that involve the accumulation of amyloid β (Aβ) plaques and hyperphosphorylated tau tangles. These hallmark protein aggregates initiate widespread neuronal damage, synaptic dysfunction, and eventually neuronal loss, leading to brain atrophy and impaired neural networks (reviewed in [63]). Next to these classical pathological features, numerous studies highlight a central role of neuroinflammation, vascular dysfunction, and mitochondrial impairment, in AD pathology (reviewed in [64]). Moreover, neural stem cell (NSC) dysfunction is associated with AD [65–69], with NSC proliferation, differentiation, and migration being disrupted by Aβ and tau proteins [70, 71].

Metabolic disturbances are increasingly recognized as key contributors to AD pathology, highlighted by significant alterations in brain lipids in AD patients and genetic risk factors associated with lipid metabolism, including *APOE*, *TREM2*, *APOJ*, *PICALM*, *ABCA1*, and *ABCA7* (reviewed in [72]). Notably, few studies found that SCD1 activity and expression is increased in plasma and brains of AD patients, and is associated with disease progression and decreased cognitive performance [73–77]. In support of enhanced SCD1 activity, post-mortem AD brains and triple-transgenic AD (3xTg-AD) mice demonstrate an accumulation of MUFA-enriched TGs within ependymal cells in the subventricular zone (SVZ) lining the ventricles, with the majority of TGs being enriched in SCD1-derived MUFAs such as OA [78]. This increase in MUFA-enriched TGs in SVZ ependymal cells was found to perturb NSC proliferation in the SVZ in a paracrine manner via soluble mediators (Fig. 1). Locally increasing OA in wild-type mice recapitulated the AD-associated ependymal TG phenotype and impaired NSC expansion. Mechanistically, OA inhibited NSC expansion by promoting hyperactivation of the AKT signaling pathway, which is consistent with SCD1 driving AKT Ser473 phosphorylation and activation in cancer cells [79, 80]. In support of the disease-promoting impact of SCD1-generated MUFAs in AD, intraventricular infusion with an SCD1 inhibitor reduced the accumulation of MUFA-rich TGs and rescued early NSC dysfunction in periventricular and hippocampal regions in pre-symptomatic 3xTg-AD mice [78]. The latter findings point towards a key role of SCD1 in controlling TG accumulation and SVZ NSC dysfunction in AD, and argues for SCD1 inhibitors being a promising therapeutic tool to attenuate early pathological hallmarks in AD. Nevertheless, observed disturbances in SVZ lipid metabolism and the impact on NSC activity also raise a number of mechanistic questions [81]. First, the root cause of the disturbances in FA metabolism and subsequent NSC dysfunction in 3xTg-AD mice remains enigmatic. It could stem from AD genetic mutations directly, represent a non-specific response to the stress AD imposes on the brain, or serve as a secondary consequence of other AD-related pathologies. With respect to the latter, although Hamilton et al. showed that impairments in neurogenesis in the 3xTg-AD mouse model occur prior to the accumulation of Aβ and tau tangles [82], other studies found that Aβ and tau can interfere with NSC function and neurogenesis [70, 71]. Hence, it remains unclear whether the disturbances in FA metabolism and subsequent NSC dysfunction are linked to Aβ and tau pathology, highlighting the need for further research. Secondly, the cellular targets and molecular mechanisms of the SCD1 inhibitor responsible for alleviating neurogenic defects in 3xTg-AD mice remain unclear. In this respect, despite the adverse effects of heightened OA levels and SCD1 activity on NSC physiology in the SVZ, OA also plays a pivotal role in promoting NSC survival within the dentate gyrus and can instigate hippocampal NSC neurogenesis [83]. This duality underscores the complexity of lipid metabolism’s impact on NSCs in various brain regions during disease progression. Lastly, the specific FA species responsible for perturbed NSC physiology in the SVZ during AD remain to be conclusively identified. While OA may contribute significantly, it is possible that the disruption also involves downstream PUFAs, or a combination of both MUFA and PUFA species.

In a follow-up study, the authors demonstrated that SCD1 inhibition also reverses deficits in spatial learning and memory in symptomatic 3xTg-AD mice [84]. Here, improved cognitive performance was associated with a reduced inflammatory activation of microglia and improved dendritic spine number and structure. When considering alterations in microglial function in AD, an intriguing parallel emerges with MS. Specifically, akin to the uptake of myelin by MS-associated microglia [38], the endocytosis of Aβ elevates SCD1 expression in microglia [85], and absence of SCD1 triggers an advantageous microglia phenotype in both AD and MS (Fig. 1). Interestingly, SCD1 inhibition did not impact NSC activity in symptomatic 3xTg-AD mice, nor did it impact other key AD hallmarks such as Aβ accumulation, tau aggregation, or neuronal loss. Although the exact molecular mechanisms behind this discrepancy remain unclear, previous research has shown that synaptic loss and memory can be restored in AD mouse models without a reduction in Aβ levels [86–88]. It is worth noting that the authors showed that SCD1 inhibition substantially reduced *Mhc-I* gene expression in activated microglia within 3xTg-AD mice [84]. Given the association of MHC-I with synaptic pruning, learning, and memory [89, 90], future investigations should aim to clarify whether SCD1 inhibition offers protection in late-stage AD by modulating MHC-I signaling in microglia. In summary, these studies suggest that SCD1 inhibition holds therapeutic potential for AD, but further research is needed to determine whether it operates through a single mechanism and cell type, or multiple mechanisms across different cell types.

### Parkinson’s disease

PD is a progressive neurodegenerative disorder characterized by the loss of dopamine-producing neurons in the substantia nigra, leading to impaired motor function. A key pathological hallmark of PD is the accumulation of Lewy bodies, which are intracellular aggregates composed predominantly of misfolded α-synuclein (α-syn). Under normal physiological conditions, α-syn is involved in synaptic function and neurotransmitter release. However, in PD, genetic mutations and environmental factors promote the abnormal aggregation of α-syn in neurons and possibly its transmission between cells, resulting in widespread synaptic dysfunction and progressive neuronal loss (reviewed in [91]). The proposed consequences of α-syn dyshomeostasis range from disrupted mitochondrial function to impaired ER-Golgi and synaptic vesicle trafficking, hampered plasma membrane integrity, reduced protein degradation, and dysregulated inflammatory responses (reviewed in [92]).

Similar to X-ALD, MS, and AD, ample evidence points towards disturbances in lipid metabolism being pathological characteristic and potentially even driver of PD disease progression. For instance, large-scale sequencing studies of PD patients have identified several risk genes related to lipid metabolism, including *GBA1*, *ASAH1*, *SMPD1*, and *SREBF1*, which are involved in the synthesis and metabolism of ceramides, sphingomyelin, cholesterol, and other major lipid classes. Furthermore, sporadic PD patients exhibit significant alterations in their brain lipidome (reviewed in [93]). Notably, by using models for α-syn dyshomeostasis, a group of neurodegenerative diseases characterized by the abnormal accumulation of α-syn in the brain, SCD1 was identified as a potential therapeutic target for PD. Employing a cellular model expressing α-syn 3 K::YFP, Imberdis et al. screened a library of 1902 compounds for their ability to lower α-syn inclusion burden, and found inhibitors of SCD as the most compelling hits [94]. Accordingly, Vincent et al. applied a phenotypic screening and target identification platform in which human α-syn is expressed in yeast under control of the galactose-inducible GAL1 promoter, and found a series of 1,2,4-oxadiazoles to protect from α-syn toxicity by inhibiting SCD1 [95]. These findings were confirmed in human iPSC-derived cortical neurons, where they showed that pharmacological inhibition of SCD1 using CAY10566 protects from α-syn toxicity. Different mechanisms for this protective effect of SCD1-inhibition were proposed by the authors. First, SCD1 inhibition was postulated to mitigate the disruption of vesicle trafficking induced by α-syn (Fig. 1). Second, SCD1 inhibition was suggested to alleviate pathogenic interactions of α-syn with membranes (Fig. 1). With respect to the latter, distinct α-syn species, such as a-syn oligomers, can interact with and disrupt lipid packing and membrane integrity [96].

In support of a key role of SCD1 in PD pathology, Fanning et al. demonstrated that human α-syn-expressing yeast have increased levels of diacylglycerols (DGs), TGs, and MUFAs, in particular SCD1-derived OA [97]. Genetic and biochemical analyses further revealed that increased OA production and DG accumulation in the ER enhanced α-syn toxicity, but only when these lipids were not converted to TGs and incorporated in lipid droplets (Fig. 1). The metabolic and toxic disturbances associated with pathogenic α-syn expression were validated in rat cortical neurons, human iPSC-derived neurons, and human ESC-derived neurons carrying the PD-causing E46K mutation. Consistent with SCD1 activity underpinning increased OA levels, pharmacological inhibitors and genetic knockdown of SCD1 reduced OA levels and attenuated α-syn toxicity in human neuroblastoma cells. By using a *C. elegans* model that expresses α-syn in dopaminergic neurons, the authors further showed that *Scd1*-deficiency protected from α-syn neurotoxicity in vivo. On a molecular level, OA was found to enhance the formation of aggregation-prone α-syn monomers in *C. elegans*, leading to heightened membrane association, gradual membrane sequestration into α-syn aggregates, and neurotoxicity.

Several other studies have identified and validated the pathogenic role of SCD in PD. For instance, the brain-penetrant SCD inhibitor 5b was demonstrated to be protective in the 3 K mouse model, in which mice have an amplified familial PD α-syn mutation (E35K + E46K + E61K [“3K”]), making α-syn more prone to aggregation [98, 99]. Inhibition of SCD by 5b was found to improve resting tremor and progressive motor decline, increase the formation of cortical aggregation-resistant α-syn species, and decrease proteinase K-resistant lipid‐rich aggregates in this model. Similar outcomes were observed in 3 K mice bred on a *Scd1* knockout background, highlighting the key role of SCD1 in PD pathology and suggesting that SCD1 is the primary target of 5b. Furthermore, SCD inhibition fully restored cell growth in neuroblastoma cells with doxycycline-inducible α-syn-3 K::YFP expression, while not reducing cell growth in the absence of doxycycline [100]. Finally, in an independent study using a similar model, SCD inhibitors were also found to reduce the formation of α-syn accumulations [101]. Knockdown experiments further demonstrated that a reduction of both *SCD1* and *SCD5* reduces α-syn accumulations, highlighting both enzymes as putative drug targets [101]. Overall, these studies underscore the detrimental impact of SCD on PD pathology; however, further research is necessary to validate the relative importance of the proposed underlying mechanisms, which include alterations in vesicle trafficking, pathogenic interactions of α-syn with membranes, and an increase in the formation of aggregation-prone α-syn. Also, caution is warranted when studying SCD1 inhibition in α-syn models, as there are some important experimental caveats. For instance, SCD1 inhibitors are established to be toxic to early human and rat neuron in vitro cultures, while displaying minimal toxicity to late cultures [101]. A reduced dependency on MUFAs and OA as cultures mature could underpin this finding, which is consistent with the decreasing expression of *SCD1* in primary neuron cultures through time [101]. Furthermore, exogenous α-syn levels can be suppressed by high SCD1 inhibitor concentrations in amplified familial α-syn E46K models, thereby obscuring the true effect size [101].

In response to findings from preclinical models, a phase 1 clinical trial (NL72549.056.20) was conducted to evaluate the impact of the SCD inhibitor YTX-7739 on PD using a randomized, double-blind, placebo-controlled design. The trial included both single- and multiple-ascending doses administered to patients with mild-to-moderate PD and healthy volunteers. No serious adverse events were reported and favorable pharmacokinetics and pharmacodynamics were demonstrated. Mild to moderate adverse events included increased PD symptoms, lower back pain, headache, and myalgia. In the treatment group, certain adverse events —such as myalgia and dry eye—occurred more frequently than in the placebo group, while others, like orthostatic hypotension and fatigue, were higher in the placebo group. After 28 days of treatment with a once-daily 20 mg dose, YTX-7739 reduced the fatty acid desaturation index by approximately 20–40%. Importantly, the drug was also shown to cross the blood-brain barrier, as indicated by target engagement in cerebrospinal fluid [102].

It is intriguing to observe that numerous PD-associated mutations are associated with lipid metabolism, notably including *SREBP1*, a key transcriptional activator of SCD1 [103, 104]. This connection raises the possibility that SCD1 inhibition might not only be a promising therapeutic avenue for PD, but also that genetic predisposition to PD could be linked to intrinsic changes in *SCD1* expression or activity. Yet, whether PD-associated mutations directly contribute to altered *SCD1* expression or activity remains uncertain and warrants further investigation.

### Other neurological disorders

Alongside playing a role in MS, X-ALD, AD, and PD pathology, significant connections between SCD1 and other neurological disorders exist. Notably, due to the protective effects of SCD1 inhibition observed in models of synucleinopathies, SCD1 may serve as a promising therapeutic target for other diseases characterized by pathogenic α-syn accumulations, such as dementia with Lewy bodies (DLB) and multiple system atrophy. Interestingly, in addition to pathogenic α-syn accumulations, DLB is characterized by early microglial activation, occurring prior to cognitive decline, along with elevated levels of IL-2, a key marker of T cell activation [105]. Given SCD1’s crucial role in dampening microglial activation and limiting effector T cell activation by enhancing Treg formation, its inhibition could offer a promising therapeutic strategy to reduce neuroinflammation in DLB and potentially other inflammatory synucleinopathies.

Huntington’s disease (HD) is also closely linked to lipid dysregulation, including significant alterations in FA metabolism (reviewed in [106]). HD is caused by mutations in the gene encoding the huntingtin protein (*HTT*), leading to the oligomerization and aggregation of Htt, ultimately resulting in neuronal death [106]. By using R6/2 HD transgenic mice, Valenza et al. demonstrated that disruptions in lipid biosynthesis are, at least partially, attributed to a mutant Htt-dependent reduction in SREBP activity [107]. As SREBP1 serves as a key transcriptional activator of SCD1 [103, 104], future studies should aim to define the causality of this signaling axis in driving lipid disturbances and the progression of HD pathology. HD has also been linked to impaired autophagy, which contributes to the accumulation of Htt aggregates [108]. Given SCD1’s established connection with autophagy (see Sect. 4.6), inhibiting SCD1 may exert protective effects in HD pathology through the induction of autophagy.

Finally, akin to AD, frontotemporal dementia (FTD), a group of disorders caused by progressive nerve cell loss in the brain’s frontal lobes, is characterized by extensive microglial activation and lipid disturbances [109]. Hence, while direct evidence is lacking, SCD1 inhibition could hold promise in suppressing pathological microglia activation in FTD. Overall, substantial evidence suggests a link between SCD1 and neurodegenerative disorders other than MS, AD, PD, and X-ALD.

## Therapeutic implications and future perspectives

Recent studies highlight the potential of SCD inhibition as a promising therapeutic avenue for alleviating inflammation and neurodegeneration, and promoting repair in CNS disorders such as MS, AD, and PD. However, several critical considerations demand thorough examination.

### Adverse side-effect associated with SCD inhibition

Although numerous SCD inhibitors have been developed, only a few have progressed to clinical trials, primarily for the treatment of type 2 diabetes [110–112], and one for Parkinson’s disease [102]. The limited number of clinical trials for SCD inhibitors is likely a result of the undesirable side effects associated with their use. Specifically, both genetic SCD1 deficiency and pharmacological inhibition of SCD result in a cutaneous phenotype characterized by dry skin, alopecia, and sebocyte hypoplasia [113–115]. Absence of SCD1 further leads to extreme cold sensitivity, with *Scd1*^−/−^ mice unable to survive longer than 2–4 h when exposed to a 4 °C environment [116]. Unlike liver-specific *Scd1*-deficiency [117], skin-specific deletion of *Scd1* recapitulates the cutaneous and thermogenic abnormalities observed in *Scd1*-deficient mice [118]. These findings point towards the importance of SCD1 in maintaining skin integrity and body temperature, and argue for inhibitors that do not target skin SCD1 being a therapeutically interesting option in neurodegenerative disorders. However, it should be noted that, akin to *Scd1*^−/−^ mice, skin-specific *Scd1*^−/−^ mice are lean and protected from diet-induced obesity and insulin resistance [119]. Given that obesity and insulin resistance are established risk factors for neurodegenerative diseases such as MS, AD, and PD [119–121], the use of SCD1 inhibitors that do not target skin SCD1 could potentially undermine the neuroprotective effects of SCD1 inhibition on these metabolic parameters. Alongside the abovementioned adverse effects, untargeted and targeted SCD1 inhibition could increase susceptibility to the development of acute colitis and lead to cellular ER stress [122, 123]. With respect to the latter, pancreatic β cells are particularly susceptible to SFA-induced ER stress after SCD1 inhibition [124, 125], which may be accelerated in individuals exposed to a Western diet rich in SFAs. In summary, the cumulative adverse effects, coupled with the uncertainties surrounding the isoform specificity of SCD1 inhibitors, particularly concerning SCD5 (see Sect. 4.2), are probable explanations for the limited clinical efficacy or significance of SCD1 inhibitors in human diseases. Future studies should investigate whether lower concentrations that achieve partial SCD or SCD1 inhibition can provide similar protective effects with fewer adverse events. Additionally, targeted CNS delivery through nanoparticles, liposomes, or BBB-specific receptor targeting could offer a promising approach for more precise SCD or SCD1 inhibition in the brain.

### SCD isoform-specific functions in neurological disorders

To date, a plethora of SCD inhibitors have been developed, including compounds with high efficacy in reducing SCD1 activity such as CAY10566 and YTX-7739. It is however essential to note that many so-called SCD1 inhibitors also impact the activity of the other human SCD isoform, SCD5, albeit to a lower degree [14]. Hence, it is important to evaluate the role of SCD5 in the healthy and diseased CNS as well. Since human SCD5 is highly abundant in the brain compared to peripheral tissues, targeting SCD5 could offer a more selective approach to inhibit SCD activity in the CNS with limited adverse effects in other tissues [9]. While the catalytic and metabolic roles and regulation of SCD1 have been extensively studied, the properties of SCD5 and its relevance in the CNS are only poorly characterized. In neurons, SCD5 has been shown to govern the initial phases of neuronal differentiation, with constitutive expression of this desaturase strongly impairing human neuronal proliferation and differentiation [126]. SCD5-mediated attenuation of phosphorylation and activation of EGFR and its downstream signaling molecules ERK and Akt, which play central roles in neuronal replication and functional maturation, was demonstrated to partially contribute to this effect. Interestingly, SCD1 activity is required for the full induction of Akt and ERK pathways [127], highlighting that human SCD isoforms may exhibit opposing functions in certain human cell types. Given the crucial role of SCD5 in neuronal proliferation and differentiation, pathological alterations in the CNS might be linked to this enzyme. Accordingly, elevated mRNA levels of *SCD5* were found in the brains of AD patients [74]. Furthermore, *SCD5* is recognized as a susceptibility gene for the development of depression, schizophrenia, and bipolar disorders, three clinical entities that are considered to be genetically connected [128]. Yet, evidence on the mechanisms by which SCD5 contributes to these pathologies is currently lacking and requires further investigation. Finally, as discussed in Sect. 4.3, knockdown of both SCD1 and SCD5 reduces the accumulation of α-syn in a neuroblastoma model [101], suggesting that, next to SCD1, SCD5 could represent an interesting therapeutic target for synucleinopathies as well. All in all, since current pharmacological modulators likely target both the SCD1 and SCD5 isoform, it is challenging to determine which isoform is responsible for the protective effects of SCD inhibition in neurodegenerative disorders. Therefore, further genetic research is needed to clarify this issue.

### Common and uncommon effects of SCD1 inhibition on neurological disorders

As outlined in this review, ample evidence underscores an important, although divergent, role for SCD1 in different neurological disorders. While SCD1 inhibition holds therapeutic potential in neurological disorders such as MS, AD, and PD [38, 73–77, 101], absence of SCD1 seems to be detrimental in X-ALD, as demonstrated by CRISPR-mediated knockout of SCD1 mimicking the motor phenotype observed in *Abcd1* zebrafish mutants [19]. This discrepancy might stem from inherent variations in the disease-associated expression and activity of SCD1. Concerning the latter, heightened SCD1 abundance is associated with disease progression in MS, AD, and PD [38, 73–77, 101], while X-ALD patients show an accumulation of saturated as compared to unsaturated VLCFAs [129], suggestive of reduced SCD1 activity. Alternatively, or concurrently, the disparity might originate from the fact that amino acid comparisons reveal a closer resemblance between zebrafish SCD homologues (such as SCD and SCDd) and human SCD5, as opposed to the similarity observed with mouse and human SCD1^135^. This suggests potential functional divergence among SCD isoforms, hinting that absence of SCD5, rather than SCD1, would exacerbate X-ALD pathology. In light of functional divergence among SCD isoforms, while *Scd1*^−/−^ mice are protected from diet-induced obesity, *Scd*-deficient zebrafish display significant ectopic visceral fat accumulation in adult stages [130]. Lastly, discrepancies in the therapeutic efficacy of SCD1 inhibitors in neurological disorders may arise from differences in the affected cell types and brain regions. Hence, understanding these nuances is crucial for unraveling the complexities of SCD1 modulation in X-ALD.

In addition to the abovementioned discrepancy, common mechanisms of SCD1 inhibition emerge across different neurodegenerative disease. For instance, in both MS and AD, the uptake of myelin or Aβ, respectively, leads to the induction of SCD1 and subsequently promotes the formation of an inflammatory, disease-promoting microglia phenotype [38, 84, 85]. With SCD1 promoting effector T cell activity at the expense of immunosuppressive Tregs as well [46], SCD1 may act as a common driver of harmful immune responses across various inflammatory neurodegenerative disorders. SCD1 inhibition also exerts protective effects through reducing MUFA-induced harmful changes in non-immune cell physiology in PD and AD. In AD, increased SCD1 activity and the concomitant formation of OA-enriched TGs in ependymal cells was found to perturb NSC proliferation [78], while in PD, enhanced SCD1 activity and OA formation enhance α-syn toxicity in neurons [94, 95, 97, 98, 100, 101]. Based on these commonalties, we postulate that targeting SCD1 represents a promising therapeutic strategy for addressing shared pathogenic mechanisms across neurodegenerative disorders, with the notable exception of disorders characterized by extensive VLCFA accumulation such as X-ALD.

### Exogenous SFAs may impair the benign impact of SCD1 inhibitors in neurological disorders

SCD1 plays a pivotal role as a regulator of peripheral metabolic disorders, including atherosclerosis, displaying a nuanced interplay of both favorable and adverse effects [131–133]. The repercussions of alterations in SCD1 activity within these pathologies appear to hinge not only on cellular and tissue-specific functions, but also on exogenous (dietary-derived) SFAs. With respect to the latter, *Scd1* deficiency combined with a Western diet rich in SFAs, rather than a high-cholesterol diet, significantly worsens the formation of atherosclerotic plaques in preclinical models [131–133]. Hence, it is tempting to speculate that an excess of SFAs, facilitated by exogenous supplementation in conjunction with SCD1 inhibition or deficiency, may potentially surpass a critical threshold, thereby amplifying SFA-induced toxicity. Consequently, investigating whether a Western diet, enriched in SFAs, renders SCD1 inhibitors ineffective or even harmful in neurodegenerative disorders, could yield valuable insights. Simultaneously, given that SCD1 abundance is highly sensitive to dietary factors (reviewed in [134]), exploring whether the impact of diet on neurological disorders is contingent on changes in SCD1 expression becomes of paramount interest. For example, obesity and adherence to a Western diet are associated with significantly increased expression and activity of SCD1, predominantly in the liver [135–137]. Conversely, considering that DHA inhibits *Scd1* expression [138], evaluating whether diet-induced variations in SCD1 underlie the benign and harmful properties of dietary interventions in neurological disorders would be a relevant avenue for further investigation. In this respect, a growing body of epidemiological, preclinical, and observational studies suggest that Western diets and omega-3 PUFA supplementation are associated with disease progression and regression of neurological disorders, respectively [139–143].

### Context-dependent impact of SFAs and MUFAs on neurological disorders

While SFAs are commonly considered lipotoxic and pro-inflammatory, MUFAs are recognized for their anti-inflammatory properties and their ability to counteract SFA-induced lipotoxicity [1]. Paradoxically, this review sheds light on the fact that pharmacological inhibition and genetic deficiency of SCD1 improves disease pathology in AD, MS and PD, despite increasing SFA levels. This incongruity begs the question what drives the benign and harmful properties of MUFAs and SFAs. We postulate that the outcomes of alterations in cellular SFA and MUFAs levels are likely multifaceted. First, variations in cellular responses to SFAs and MUFAs can be attributed to cell- and context-dependent differences. This notion aligns with the diverse fates of these FAs, encompassing their integration into membrane lipids, storage within lipid droplets, acting as protein modifiers, or undergoing mitochondrial β-oxidation [1]. In support of this concept, divergent cell types display varying resilience to SFA-induced ER stress, and the transcriptional response to this stress can be modified under diverse metabolic and pathological conditions [144–149]. In a related context, ABCA1 destabilization by MUFAs in macrophages is more likely to become inflammatory in those cells that rely on lipid efflux to protect themselves from lipotoxicity, such as myelin-containing phagocytes in demyelinating disorders like MS [38]. Accordingly, *Abca1*-deficiency in non-lipid-loaded macrophages induces a pro-angiogenic phenotype, characterized by a reduced expression of inflammatory mediators [150]. Finally, alterations in cellular physiology are also reported to impact the channeling of FAs within cells. For instance, pro-inflammatory macrophages exhibit higher accumulation of exogenous FAs within TGs and CEs, while anti-inflammatory macrophages show increased enrichment of FAs within glycerophospholipids, ether lipids, and sphingolipids [151]. The molecular underpinnings of these variations and the significance of these alterations in preserving the functional phenotype remain elusive. Alongside cell- and context-dependent changes, the source of FAs, whether endogenously synthesized or exogenously administered, may contribute to their harmful or beneficial impact on cell physiology. For example, endogenously synthesized and exogenously administrated FAs are reported to show a divergent positional distribution in TGs and impact cellular function differently, thereby having a divergent impact on lysosomal acidity and MHC class II expression [152, 153]. All in all, this intricate web of interactions emphasizes the need for a comprehensive understanding of cellular and conditional factors influencing the diverse effects of SFAs and MUFAs on cellular physiology and pathology.

### SCD1 and autophagic processes in the brain

SCD1, in addition to or as a result of its role as a rate-limiting enzyme in SFA desaturation, exerts significant influence on a diverse range of cellular processes that were not discussed in this review. For example, emerging evidence indicates an important link between SCD1 and autophagy, and dysregulation of autophagy has been implicated in multiple neurodegenerative proteinopathies, including AD, PD, and HD (reviewed in [154]). Studies conducted in *Drosophila*, *S. cerevisiae*, and mammalian cell cultures revealed that deficiency of *Desat1*, *Ole1*, and *Scd1*, respectively, impedes autophagy induction during starvation, accompanied by impaired translocation of key autophagy proteins such as ULK1, P62/SQSTM1, and ATG9 to autophagosome formation sites [155–157]. Intriguingly, contradicting observations have been reported in colon and hepatocellular carcinoma cell lines in non-starved conditions, where genetic deficiency and pharmacological inhibition of SCD1 enhances autophagy by activating AMPK and subsequently suppressing mTOR signaling [158, 159]. In support of this discrepancy, the impact of elevated palmitate levels on selective autophagy of the ER may differ under starvation versus nutrient-rich conditions as well [160]. While these investigations underscore the importance of SCD1 in autophagy regulation, the precise mechanisms underlying these discrepancies as well as the importance of SCD1-mediated autophagy regulation in neurological disorders remain to be elucidated.

## Summary

Collectively, this review emphasizes the crucial role of SCD1 in contributing to the pathogenesis of diverse neurodegenerative and neuroinflammatory disorders, thereby highlighting the therapeutic potential of SCD1 modulation in conditions such as MS, AD, and PD. In this regard, early-phase 1 clinical trials in PD using YTX-7739 have shown highly promising results, indicating the feasibility of SCD modulation as a therapeutic strategy. However, despite these promising prospects, it remains essential to carefully consider potential hurdles related to SCD inhibitor specificity and associated side-effects. Moreover, given the detrimental impact of SCD1 deficiency on X-ALD, careful consideration of cell- and tissue-specific effects, along with disease- and context-dependent differences, is essential for advancing the clinical application of SCD1 modulators.

## Data Availability

Not applicable.
